# Implications of COVID-19 control measures for diet and physical activity, and lessons for addressing other pandemics facing rapidly urbanising countries

**DOI:** 10.1080/16549716.2020.1810415

**Published:** 2020-09-01

**Authors:** Tolu Oni, Lisa K. Micklesfield, Pamela Wadende, Charles O. Obonyo, James Woodcock, Ebele R. I. Mogo, Feyisayo A. Odunitan-Wayas, Felix Assah, Lambed Tatah, Louise Foley, Clarisse Mapa-Tassou, Divya Bhagtani, Amy Weimann, Camille Mba, Nigel Unwin, Anna Brugulat-Panés, Karen J. Hofman, Joanne Smith, Marshall Tulloch-Reid, Agnes Erzse, Maylene Shung-King, Estelle V. Lambert, Nicholas J. Wareham

**Affiliations:** a MRC Epidemiology Unit, University of Cambridge, Cambridge, UK; b Research Initiative for Cities Health and Equity (RICHE), School of Public Health and Family Medicine, University of Cape Town, Cape Town, South Africa; c South African MRC/Wits Developmental Pathways for Health Research Unit (DPHRU, University of Witwatersrand, Johannesburg, South Africa; d School of Education and Human Resource Development, Kisii University, Kisii, Kenya; e Centre for Global Health Research, Kenya Medical Research Institute, Kisusmu, Kenya; f Research Centre for Health through Physical Activity, Lifestyle and Sport (HPALS, University of Cape Town, Cape Town, South Africa; g Health of Populations in Transition (HoPiT, University of Yaoundé, Yaounde, Cameroon; h South African MRC Centre for Health Economics and Decision Science (PRICELESS SA), Faculty of Health Sciences, School of Public Health, University of Witwatersrand, Johannesburg, South Africa; i Caribbean Institute for Health Research, The University of West Indies, Kingston, Jamaica

**Keywords:** Diet, physical activity, COVID-19, urbanisation, non-communicable diseases, informal settlements, policy

## Abstract

At the time of writing, it is unclear how the COVID-19 pandemic will play out in rapidly urbanising regions of the world. In these regions, the realities of large overcrowded informal settlements, a high burden of infectious and non-communicable diseases, as well as malnutrition and precarity of livelihoods, have raised added concerns about the potential impact of the COVID-19 pandemic in these contexts. COVID-19 infection control measures have been shown to have some effects in slowing down the progress of the pandemic, effectively buying time to prepare the healthcare system. However, there has been less of a focus on the indirect impacts of these measures on health behaviours and the consequent health risks, particularly in the most vulnerable. In this current debate piece, focusing on two of the four risk factors that contribute to >80% of the NCD burden, we consider the possible ways that the restrictions put in place to control the pandemic, have the potential to impact on dietary and physical activity behaviours and their determinants. By considering mitigation responses implemented by governments in several LMIC cities, we identify key lessons that highlight the potential of economic, political, food and built environment sectors, mobilised during the pandemic, to retain health as a priority beyond the context of pandemic response. Such whole-of society approaches are feasible and necessary to support equitable healthy eating and active living required to address other epidemics and to lower the baseline need for healthcare in the long term.

In rapidly urbanising low- and middle-income countries (LMIC), the realities of large overcrowded informal settlements, a double burden of infectious and non-communicable diseases (NCDs), malnutrition and the precarity of livelihoods [1] have raised added concerns about the potential impact of the COVID-19 pandemic. Control measures implemented in these settings include closing of national and state/county borders, and schools, restricted movement (including frequency of shopping), working from home, provision of water in informal settlements for hand hygiene, physical distancing and the banning of large gatherings. These measures have been shown to slow down transmission, buying healthcare systems preparation time [2]. However, there has been less of a focus on the indirect impacts of these measures on health behaviours and consequently health risks, particularly in the most vulnerable.

These restrictions have the potential to impact NCD risk factors such as dietary intake and physical activity behaviours [3], by limiting access to healthy foods and to environments conducive to physical activity. While many of the interventions to curb the pandemic are intended to be temporary, they have exposed existing inequities in disease vulnerability and access to care. The amplification of these inequities is resulting in poorer disease outcomes and negative impacts on livelihoods among the poor and marginalised.

This debate piece will describe how the implementation of restrictions to limit the spread of the pandemic may impact diet and physical activity behaviours, as well as their determinants. We also reflect on the experiences from mitigation interventions mobilised by several governments to respond to this pandemic, and the potential for these to be leveraged beyond COVID-19 in order to retain health as a priority.

## Impact of control measures on diet and physical activity

With the implementation of measures to prevent further spread of COVID-19 infections, LMICs are experiencing two inter-related pandemics. Our public health response to the COVID-19 pandemic may be exacerbating behaviours such as unhealthy food consumption, physical inactivity and sedentary behaviour. These aforementioned behaviours are considered the key drivers of obesity epidemic globally, and obesity is a risk factor for adverse outcomes of COVID-19 infection [4].

External dimensions of the food environment include the availability, price, vendor and product properties, and promotional information. By comparison, personal dimensions of the food environment are the accessibility, affordability, convenience and desirability of food sources and products. These food environment exposures, which vary considerably between and within high-, middle- and low-income countries, influence food choices, dietary habits, and food security [5,6].

The COVID-19-related closures of national borders threaten food supply especially in countries already facing food insecurities and those largely dependent on food imports. As a result, maintaining adequate food production, import, storage and transportation can be precarious for both external and personal dimensions of the food environment. The dynamics of purchasing and consumption of healthy food in this context, driven by limited access to fresh foods and refrigeration, has highlighted the growing wealth inequality within countries.

For many, the new reality of shelter-in-place measures has resulted in an increase in cooking and eating at home. On one hand, this presents an opportunity for preparing more healthy meals at home. However, a lack of nutritional knowledge and cooking skills, increased snacking behaviour, unavailability and increased costs of healthy foods [7], and the lack of guidelines to ensure nutritional quality of food parcels to the poor [8] may result in a shift to more unhealthy processed food consumption. This is particularly important for lower-income households without the means, or in some cases, the access to refrigeration, to stock up on fresh food supplies. In addition, school closures result in interrupted access to food for children from households that depend on school-feeding programmes, adding to the financial burden and household food insecurity.

These measures are also resulting in a staggering loss of jobs and livelihoods that further impact on people’s ability to afford healthy foods. The predominance of the informal economic sector and absence of robust social safety schemes in LMICs further compound food affordability. In these circumstances, without social welfare measures, the most vulnerable are left with no choice but to defy social distancing measures to avoid hunger.

COVID-19 control measures inevitably disrupt routine daily activities and may have positive or negative consequences on physical activity behaviours. Stay-at-home orders and curfew measures result in reduced travelling, which in many cases includes walking, potentially reducing the opportunity for physical activity. Reduced regular physical activity alone, or compounded by unhealthy eating may result in an increased risk or worsening of chronic conditions such as obesity, diabetes, hypertension and cardiovascular disease [9,10], which are increasing in prevalence in LMICs [3]. These are the very conditions that have also been associated with an increased risk of hospitalisation and death from COVID-19 [11]. Some evidence from other emergencies suggests that the deleterious effects of physical inactivity and sedentarism that begin during an outbreak may continue for some time after the end of the outbreak [12]. Conversely, this new reality may encourage more physical activity as people seek respite from being stuck at home, particularly if the reduction in traffic makes built environments more conducive to physical activity in public spaces. As such, it would be important to mitigate indirect negative consequences to encourage safe physical activity. While doing this at home would be ideal, it is important to recognise that for the majority of the urban poor, particularly in the context of informal settlements [13], this will not be practical. Furthermore, outdoor physical activity has been associated with greater enjoyment and increased likelihood of achieving the recommended levels of physical activity [14]. This highlights a critical need for guidelines on safe physical activity outdoors at the different alert levels of pandemic response.

## Opportunities for systems change towards equitable health creation

Many governments in LMICs have implemented measures to minimise COVID-19 risk while working to address underlying social inequities that further increase vulnerability to disease. In so doing, the emergency has highlighted the possibility of previously considered impossible or unfeasible actions [15].

We note the following reflections from these pandemic responses that could be applied to current and future epidemics and pandemics to support equitable access to healthy diets and physical activity:

1. Wide-ranging multisectoral action to reduce inequalities is possible at pace when there is social and political will. The response to the COVID-19 pandemic has seen unprecedented multisectoral action compared to responses to the ongoing obesity pandemic. Of note, governments have led coordination of multisectoral responses, removing bureaucratic processes that could hinder support for vulnerable communities.
  For example, in Kenya, the Ministry of Agriculture has partnered with county governors to aggregate information on the volumes and prices of staples and nutritious foods in order to ensure affordability [16]. County governors have also prioritized identifying vulnerable families to ameliorate food insecurity [17]. Beyond this pandemic, mapping access to nutritious foods and governing their equitable distribution can be utilised to improve dietary behaviours, especially for the urban poor. The implementation of risk-containment strategies has also required effective partnerships at the community level, with community health volunteers and grassroots organizations constituting the last mile of distribution of health information, rapid testing, healthy food and other supplies. Beyond an acute emergency response, continued collaboration with these same partners can ensure that physical activity and healthy diets remain accessible. In another example of government-led strategies to support food security, the State Commissioner for Agriculture in Nigeria established makeshift neighbourhood food markets in schools in Lagos which were closed due to the lock down [18]. In a mega-city where fresh food markets are often centralised in large markets, like Mile 12 market [19], this intervention brought fresh food supplies closer to neighbourhoods to mitigate the impact of the closure of centralised markets and movement restrictions across the city. In Jamaica, with the closure of international borders and the sudden loss of the demand for food from the tourist industry, the government introduced community-based cashless farmers markets and distribution of fixed priced ‘vegetable baskets’ though community organizations to reduce the waste of perishable agricultural items and help support this sector [20]. Larger food manufacturers also increased capacity for fruit preservation though production of purees and manufactured new fruit blends [21].
  Beyond this pandemic, it would be important to reflect on and evaluate the experience of these endeavours to inform longer term strategies to make fresh foods more locally available within neighbourhoods.
2. COVID-19 has revealed significant flaws in our existing urban infrastructure. These flaws include economic systems that reduce resilience to food insecurity, and streets that prioritize motorised traffic, making physical activity for leisure or travel unsafe. Understandably, the majority of emergency mitigation responses have focused on food security. However, some settings such as Bogota, Colombia have created new space for walking and cycling [22] while the Jamaican Ministry of Health and Wellness has shifted focus to television and social media-based exercise programmes [23]. Given that physical distancing measures are likely to be recommended for some time, contextually relevant research to encourage and support safe accessible physical activity for all should inform recovery plans related to the built environment.
3. There is a need for public health interventions jointly to reinforce democracy, the rights of the individual and the collective good. In the context of acute response to emergencies, varying approaches have been adopted including extensive tracing using big data and deployment of the police to enforce movement restriction measures. If rights are side-lined, these measures potentially compromise data privacy and individual rights if applied in a discriminatory manner. This highlights the importance of a rights-based approach to public health, recognising that the right to health is dependent upon other human rights (such as food, housing, information and participation) and that even well-intended enforcement could bring harms. A rights-based approach could similarly be applied to addressing the obesity pandemic. For example, governance of urban space could ensure that organizations charged with enforcing lockdown measures are thoroughly oriented in equitable governance approaches, and partner with community stakeholders to improve access to neighbourhood resources including safe spaces for physical activity. Additionally, it can include collaboration with key actors to facilitate access to space for urban agriculture for the poor.
  While the impacts of these interventions are yet to be evaluated, these examples hint at the potential for whole-of-society approaches to building stronger systems for health and lower the baseline need for healthcare. In the long term, it is vital that the economic, political, food and built environment sectors mobilised during the pandemic are encouraged (and governed) to retain health as a priority. Societies that support and enable healthy eating and active living are vital to reduce vulnerability to COVID-19 and other diseases and pandemics in the long term.

## Paper context

In urban populations with inadequate access to sanitation, food and physical activity, and growing obesity and NCD burden, COVID-19 control measures can widen health inequities. Addressing the pandemic while mitigating this vulnerability calls for a rights-based approach to governance of urban space. Multisectoral government COVID-19 responses in these cities to address social inequities highlight the feasibility of economic, political, food and built environment sectors, mobilised during the pandemic, to support healthy eating and active living.
